# Inducing lateralized phosphenes over the occipital lobe using transcranial magnetic stimulation to navigate a virtual environment

**DOI:** 10.1371/journal.pone.0249996

**Published:** 2021-04-14

**Authors:** Adonay N. Gebrehiwot, Tatsuya Kato, Kimitaka Nakazawa

**Affiliations:** 1 Department of Life Sciences, The University of Tokyo, Tokyo, Japan; 2 Japan Society for the Promotion of Science, Tokyo, Japan; University of Toronto, CANADA

## Abstract

Electrical stimulation involving visual areas of the brain produces artificial light percepts called phosphenes. These visual percepts have been extensively investigated in previous studies involving intracortical microsimulation (ICMS) and serve as the basis for developing a visual prosthesis for the blind. Although advances have been achieved, many challenges still remain with implementing a functional ICMS for visual rehabilitation purposes. Transcranial magnetic stimulation (TMS) over the primary occipital lobe offers an alternative method to produce phosphenes non-invasively. A main challenge facing blind individuals involves navigation. Within the scientific community, methods to evaluate the ability of a visual prosthesis to facilitate in navigation has been neglected. In this study, we investigate the effectiveness of evoking lateralized phosphenes to navigate a computer simulated virtual environment. More importantly, we demonstrate how virtual environments along with the development of a visual prosthesis share a mutual relationship benefiting both patients and researchers. Using two TMS devices, a pair of 40mm figure-of-eight coils were placed over each occipital hemisphere resulting in lateralized phosphene perception. Participants were tasked with making a series of left and right turns using peripheral devices depending on the visual hemifield in which a phosphene is present. If a participant was able to accurately perceive all ten phosphenes, the simulated target is able to advance and fully exit the virtual environment. Our findings demonstrate that participants can interpret lateralized phosphenes while highlighting the integration of computer based virtual environments to evaluate the capability of a visual prosthesis during navigation.

## Introduction

One of the main challenges impacting blind individuals involves the ability to navigate independently within their environment [1]. Current research that aims to rehabilitate visual perception have utilized various techniques. Such measures involve restoring damaged areas at a cellular level along the retina-geniculate-striate pathway using genome editing, stem cell transplant, optogenetics, and electrode array implants [2–8]. These developments have the potential to help blind individuals regain their independence especially while navigating. With approximately 40 million individuals impacted by blindness globally, the demand for extensive research in this area is needed [9].

Methods to evaluate the competency of a visual prosthesis in aiding a blind individual to successfully navigate through stimulation of cortical visual areas will need additional examination. Upon further investigation, a general consensus within the scientific community to assess the role of visual simulations towards the development of a visual prosthesis remains challenging [10–12]. Although the literature has poured extensive research into the technical developments involving a visual prosthesis, minimal attempts has gone into testing a devices efficiency while facilitating in navigation. Not to mention the need to address appropriate rehabilitative protocols individuals experiencing visual deficits will need in becoming familiar with any new system [13]. Such proposals must be adequate in addressing the safety needs of the individual while allowing researchers the opportunity to troubleshoot and modify potential errors within the proposed objectives [10]. One potential method that could be deployed to tackle these concerns involves the utilization of computer based virtual environments. These software can be developed to replicate situations that a blind individual may encounter on a daily basis. The goal of this study highlights how developing such computer programs can share a mutual relationship in the pursuit of developing a visual prosthesis for the blind.

Earlier studies involving electrical stimulation of the visual cortex utilized artificial light percepts called phosphenes [14–18] Investigating phosphene perception has played a vital tool for developing a cortical visual prosthesis over the years. Offering a larger surface area to activate, intracortical microsimulation (ICMS) efficiently produces phosphenes at lower currents compared to surface electrodes [16,17]. However, many challenges still remain concerning implantable electrodes. For instance, accessing critical visual areas located along the calcarine sulcus to elicit phosphenes remains problematic [19]. Cortical electrical stimulation can lead to neural tissue degeneration and even towards cell death [20]. Moreover, preventing electrode degradation overtime while optimizing functionality will be necessary in order to sustain visual resolution [21]. Visual impairments can stem from various conditions along the retina-geniculate-striate pathway, hence a prosthesis that encompasses individuals within this population will also be necessary [22,23]. The aforementioned issues are just a few points that will need to be addressed if ICMS are to become successful [22].

Transcranial magnetic stimulation (TMS), which is used most commonly as a measure to analyze cortical excitability, offers new insights towards developing a visual prosthesis. TMS over the occipital lobe is also known to produce phosphenes. More interestingly, depending on the severity of the visual impairment, blind individuals have the capacity to experience phosphenes while utilizing TMS [24]. Although these brief flashes of light do not recapture full visual perception, it may be applicable for navigational purposes. Losey [25] demonstrated these benefits while illustrating the usefulness of TMS produced phosphenes. The authors evoked phosphenes while distinguishing between sub-threshold and above-threshold intensities within one visual hemifield to signal directional responses in order for participants to navigate an avatar through a computer based virtual world.

Due to its non-invasive utilization, producing phosphenes with TMS instead of ICMS minimizes complications that can arise from surgery [26]. Investigating these methods offers a new dimension in terms of developing a visual prosthesis for the blind that could assist them in navigating within their surroundings. This method also offers a practical approach to investigate how phosphenes can be used to navigate a computer based virtual environment that is based on the topographic organization of the primary visual cortex (V1). More specifically, the proposed experimental design incorporates these anatomical fundamentals to navigate a virtual environment to initiate a series of left and right turns; a behavior experienced if one were to walk upright in a linear direction.

In the following study, we investigate the effectiveness of evoking lateralized phosphenes to navigate through a virtual environment using two TMS devices. Placing a figure-of-eight coil over each occipital hemisphere will produce contralateral phosphenes within an individual’s field of vision. Navigation is achieved through user inputs via two customized peripheral devices. Completing the objective solely relies on information transferred by localized phosphenes, indicating directional cues to successfully navigate a given virtual area. We hypothesize that lateralized phosphenes can act as effective artificial sensory inputs to complete a task involving navigation. These inputs incorporated within a computer based virtual environment could possibly advance research relevant to the development of a visual prosthesis that can effectively assist a blind individual during navigation.

## Materials and methods

### Participants

Fifteen healthy volunteers were screened for phosphenes (8 males, 7 females, M = 26.2, SD = 2.5 years). Prior to beginning, participants (Par.) were screened for visual acuity by conducting a Snellen eye exam. Information regarding participants use of glasses was also collected. All participants reported no previous neurological conditions that would prevent the use of TMS under the context of the experimental design. In order to execute the experimental design, participants were required to report lateralized phosphenes while meeting the phosphene threshold (PT). Five participants were able to perceive lateralized phosphenes in both visual hemifields, however they fell short in meeting the PT, therefore they were excluded from the study. Five participants did not report any phosphenes in either visual hemifield. The final five participants were able to achieve the criteria (3 males, 2 females, M = 25.8, SD = 1.5 years, Table 1), and were used to complete the following study. These participants were naïve to the experimental design and had no previous involvement in TMS related experiments. The study was reviewed and approved by the ethics committee at the University of Tokyo. Written consent forms were signed by all participants in congruence with the Declaration of Helsinki.

**Table 1:** Participant information.

*Participant*	*Age*	*Sex*	*Snellen Score*		*Glasses/Onset (Years)*
			*Right Eye*	*Left Eye*	
1	24	M	20/100	20/100	+/10
2	26	M	20/63	20/63	+/16
3	25	M	20/40	20/40	-
4	26	F	20/200	20/200	+/12
5	28	F	20/25	20/20	-

### Experimental equipment

Two transcranial magnetic stimulators with different stimulus configurations were used (Magstim Co., Whitland, UK, Magstim Rapid^2^, biphasic stimulator, 15k-30k Tesla, pulse-width 400ms; Magstim 200^2^, monophasic stimulator, 24k-47k Tesla, pulse-width 1ms) along with two 40mm figure-of-eight coils. The 40mm figure-of-eight coils were ideal for our experimental design. Due to its relatively small size, these coils made it possible to place two over each occipital hemisphere. The Rapid^2^ stimulator applied single pulses over the right occipital hemisphere, while the Magstim 200^2^ applied pulses over the left occipital hemisphere. Using a foot switch, the first stimulation started at 40% intensity output. If no phosphenes were present after applying three pulses, TMS intensity output was increased by increments of 5% until 100% was reached. Pulses were applied every 7 seconds as a safety precaution during the preassessment [27].

**Fig 1:**
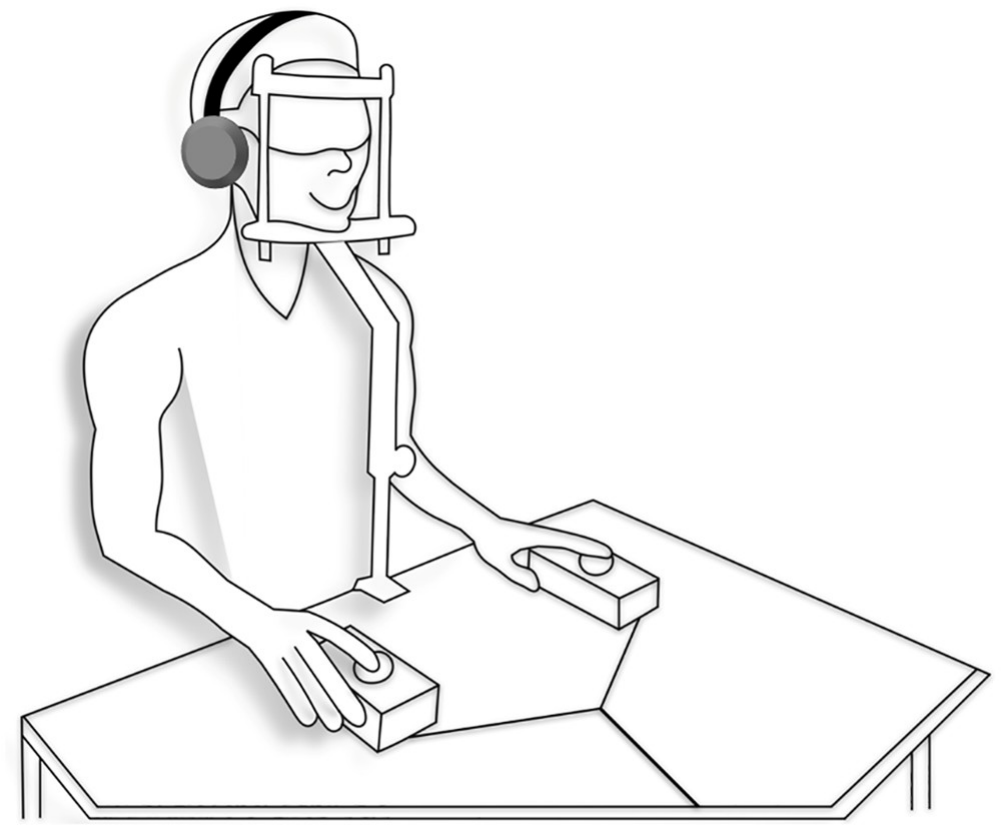
Experiment set-up. Illustration of the experimental setup with pushbuttons used to navigate throughout the virtual environments. Maintaining coil placement over the hotspot is crucial, therefore a chin-headrest was used to maximize stability.

Noise cancelling headphones were provided during the experiment, which aids in reducing auditory stimuli emanating from the TMS coils [28]. Blindfolds were also given, which help to maximize the awareness of phosphenes. Finally, to minimize any head movements during the experiment, a chin-headrest was securely attached to a table positioned in front of the participants (Fig 1). This assisted in maintaining the coil over the identified phosphene hotspot throughout the experiment, which was held individually by the two experimenters.

### Visual cortex mapping

**Fig 2:**
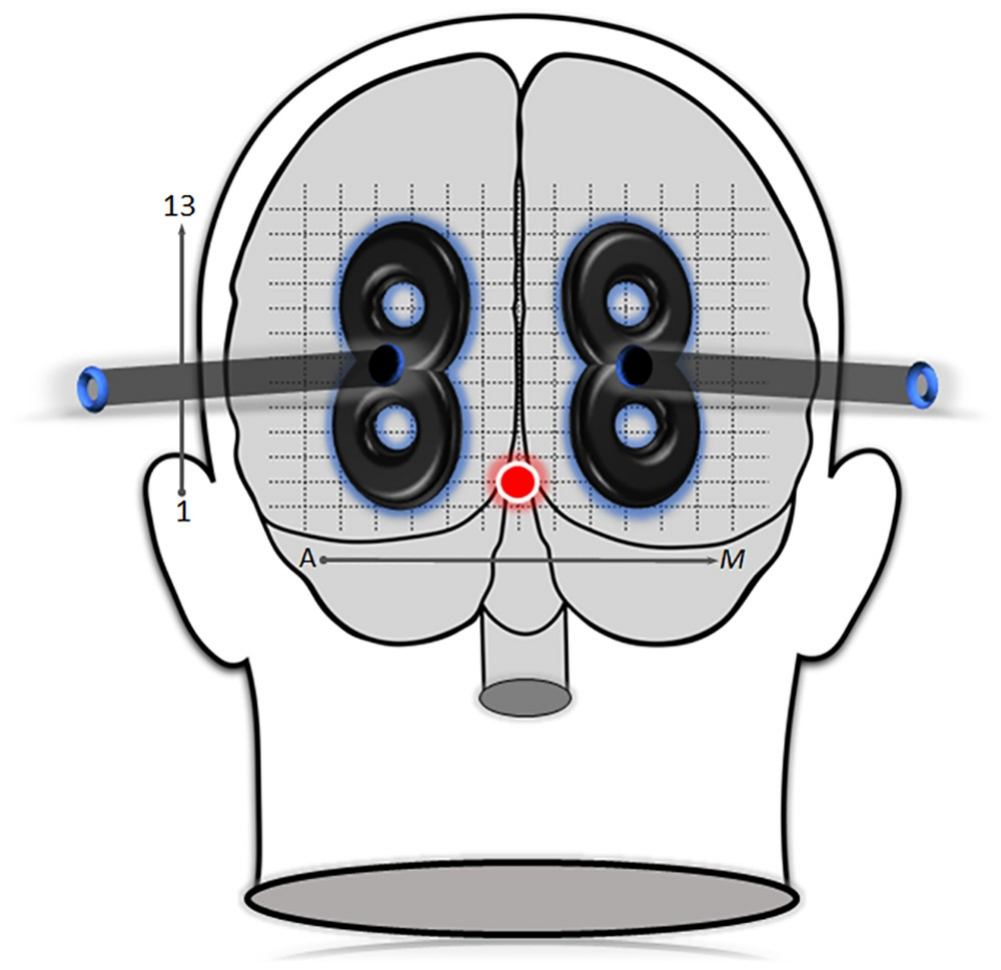
TMS mapping. Surface grid placement used to determine the phosphene hotspot. The x-axis is organized alphabetically (A-M), while the y-axis is arranged numerically (1–13). The red circle reflects the location of the inion at coordinate G-2 with two adjacent 40mm figure-of-eight coils placed over the occipital hemispheres.

A tightly fitted cotton swim cap was placed over the participant’s head with a 13x13cm surface grid marked over the occipital surface. The horizontal axis was labelled alphabetically from A-M, while the vertical axis was labeled numerically from 1–13. A red circle was drawn over G-2 indicating the inion. This location acted as a reference point for all participants (Fig 2). Seated in a dimly lit room, the initial single pulse occurred at location F-4 for the left hemisphere and at H-4 for the right hemisphere.

Before starting, participants were educated on phosphene characteristics to ensure accurate reporting. Phosphene locations will vary between individuals and can take on various geometrical shapes, lines, and colors. Phosphene documentation in Marg and Rudiak [29] was used as a template to help determine if participants were perceiving true phosphenes. Phosphene perception had to be present in the contralateral visual hemifield in relation to the stimulation site reflecting the topographic organization of the primary visual cortex. Only if these features were reported by participants were they allowed to continue on with the experiment. During the preassessment, participants were instructed to state “yes” if they perceived a phosphene after stimulation. If unsure, they were required to reply “maybe,” and a subsequent pulse was administered. If they remained quiet after a stimulation was given, this indicated “no,” and the coil was repositioned onto the next stimulation site [27]. This consisted of repositioning the coil in a randomized rostral-lateral fashion per participant [24]. At any time, participants were informed to mention any discomfort resulting in the discontinuation of the experiment. When a phosphene hotspot was determined, the outline of the coils were traced using a marker. PT is characterized as the lowest stimulation intensity where 10 out of 10 phosphenes are perceived by the participant [25]. Therefore, a total of 20 out of 20 phosphenes were required when considering for both occipital hemispheres. Reaching this criterion was needed to ensure that participants were able to navigate throughout the virtual environment to demonstrate the effectiveness of utilizing such computer programs. The experimental TMS intensity was then set to 105% of the PT in order to produce consistent phosphenes. Additionally, two things were noted: 1) surface grid coordinates and 2) phosphene shape and color (Table 2).

**Table 2:** Participant phosphene information.

*Left Hemisphere Stimulation*:						
*Participant*	*Hotspot Coordinates*	*TMS Intensity (PT × 1*.*05)*	*Phosphene Shape*	*Phosphene Color*	*Field of Vision*	
					*L*	*R*
1	F5	70	Wavy Line	White	-	+
2	F4	42	Sector	White	-	+
3	F5	43	Sector	White	-	+
4	F6	63	Flash	White	-	+
5	F4	63	Rectangle	White	-	+
*Right Hemisphere Stimulation*:						
*Participant*	*Hotspot Coordinates*	*TMS Intensity (PT × 1*.*05)*	*Phosphene Shape*	*Phosphene Color*	*Field of Vision*	
					*L*	*R*
1	H3	100	Circle	White	+	-
2	H4	64	Circle	White	+	-
3	H5	42	Sector	White	+	-
4	H6	74	Flash	White	+	-
5	H5	87	Square	White	+	-

Stimulation information including hotspot coordinates, phosphene features, and TMS intensity output over the left (Magstim 200^2^) and right (Magstim Rapid^2^) occipital lobe. PT corresponds to the phosphene threshold determined during the preassessment.

### Experimental design

A total of ten virtual environments including five experimental conditions and five control conditions were programmed. The order was randomized for every participant. Each one was designed to include ten 90° turns split evenly between left and right. The order between turns were randomly sequenced with variations in distance and velocity. Along this programmed route, a moving green circle (target) acted as the participant’s position within the virtual environment. The time at which a target travels to the next location spans between 7–15 seconds for safety measures. Implementing the virtual environment demonstrates the participants movement in space. More specifically, to highlight how phosphenes can influence someone’s ability to navigate from one place to another.

Peripheral devices comprised of two pushbuttons that were positioned in front of the participant (Fig 1). The experimental goal requires each participant to press one correct button in response to the visual hemifield in which the participant perceives a phosphene. If a button was pressed (ON-state), this event is recorded by a value of one. Oppositely, a value of zero indicates that a button was not pressed (OFF-state). These binary inputs determined the targets next move and was saved into a matrix for statistical analysis.

**Fig 3:**
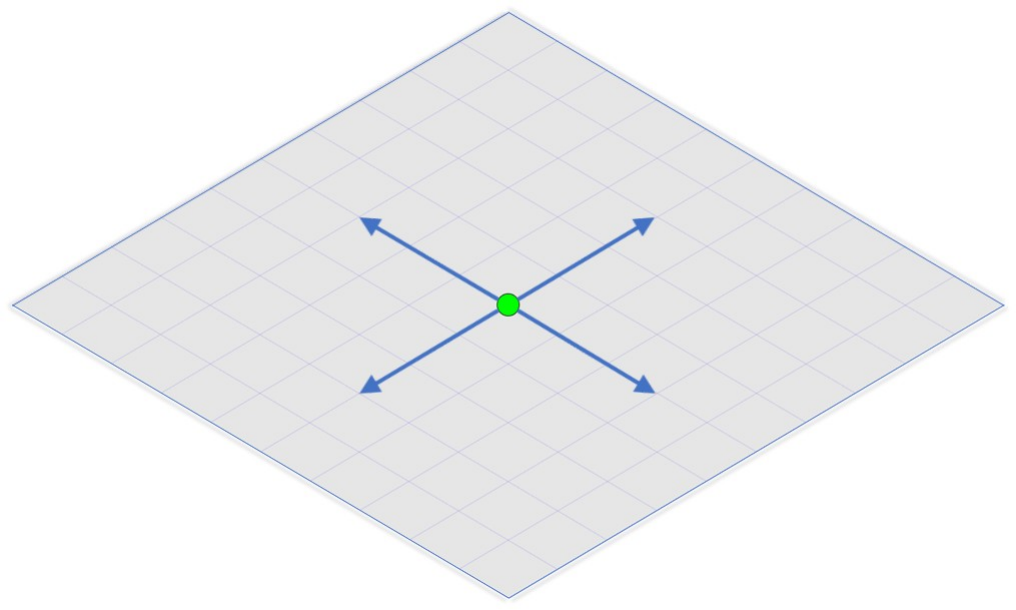
Virtual environment depiction. The participants location is represented by a green circle (target). This figure illustrates the layout of the virtual environment as displayed on the computer monitor, which requires the target to make a series of left and right turns. Once coming to a full stop, one of the two TMS coils will administer a single pulse over the participants occipital lobe depending on the programmed route (blue arrows). If participants respond correctly to the voice-automated questions that follow, the target will make a 90° turn in order to advance towards the next location. Perceiving all lateralized phosphene percepts allows the target to successfully exit.

The process is initiated when the participant hears a beep through the headphones. This event signals that the target is moving in a linear trajectory (Fig 3). At a given location, the target will come to a halt where a single pulse is administered to either the right or left occipital hemisphere depending on the programmed route, which is not displayed on the monitor. For example, if the target is required to turn left, the coil positioned over the right occipital hemisphere will apply a single pulse causing the participants to perceive a phosphene within their left field of vision. If the target is required to turn right, then the opposite occurs. In this instance, the coil positioned over the left occipital hemisphere will apply a single pulse allowing the participant to experience a phosphene within their right field of vision. The total number of TMS pulses administered equals the number of total turns, therefore participants could not exceed the maximum limit. If the maximum limit was reached at any given moment, the program was designed to exit the current session and move on to the next virtual environment.

**Fig 4:**
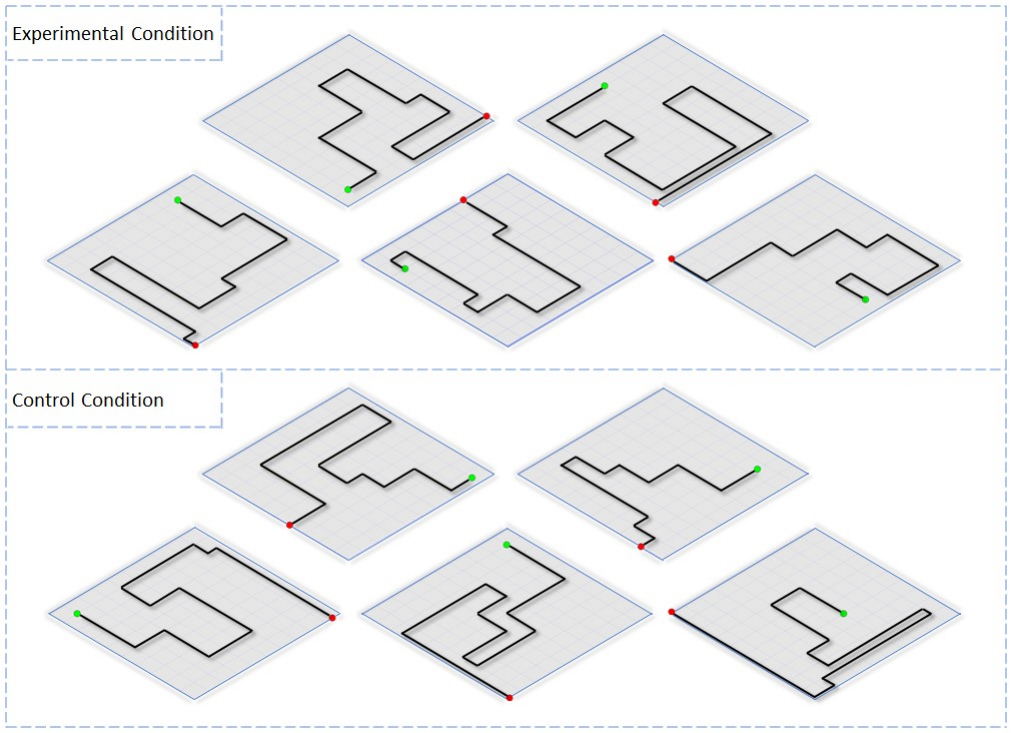
Virtual environments. Design of all ten virtual environments. The top five were assigned to the experimental condition, while the bottom five were assigned to the control condition. The green circles indicate the participants starting position before each session, while the red circles indicate the exit.

Two voice-automated sentences were programed to play following a TMS pulse. The first sentence states, “press left for no or right for yes.” Participants answered the question by pressing one of the two pushbuttons. If the left button was pressed, this meant the participant was not able to perceive a phosphene causing the target to remain at the same location. Otherwise, if the right button is pressed indicating they perceived a phosphene, participants were prompted to the second question which states, “turn left or turn right.” Again, participants answered by pressing one of the two pushbuttons. If the participant correctly presses the button that corresponds to the location of the phosphene, only then will the target advance, repeating the sequence of events described above. If ten turns are successfully made, the participant was able to accurately decipher the location of each phosphene in order to exit (Fig 4). If the wrong button (e.g., pressing the right button when a TMS pulse was applied over the right hemisphere) is pressed following the second question, the program will automatically end the session. This action was implemented to help alert the experimenters to reassess the proper TMS output intensity and hotspot coordinates, since errors in these two components can cause ipsilateral phosphene perception. Throughout the study, the following event did not occur, implying all participants were perceiving contralateral phosphenes.

### Conditions

Five virtual environments were assigned to the experimental condition (Fig 4). In theory, participants will answer “yes” that they perceived a phosphene, and accurately select the button that corresponds to the visual hemifield where the phosphene is present after the second question is played. However, if the participant does press “no,” a subsequent TMS pulse will be administered while the target remains at the same location. The session will continue until all ten TMS pulses have been applied. In this scenario, even one “no” response will result in the participant not being able to fully exit.

**Fig 5:**
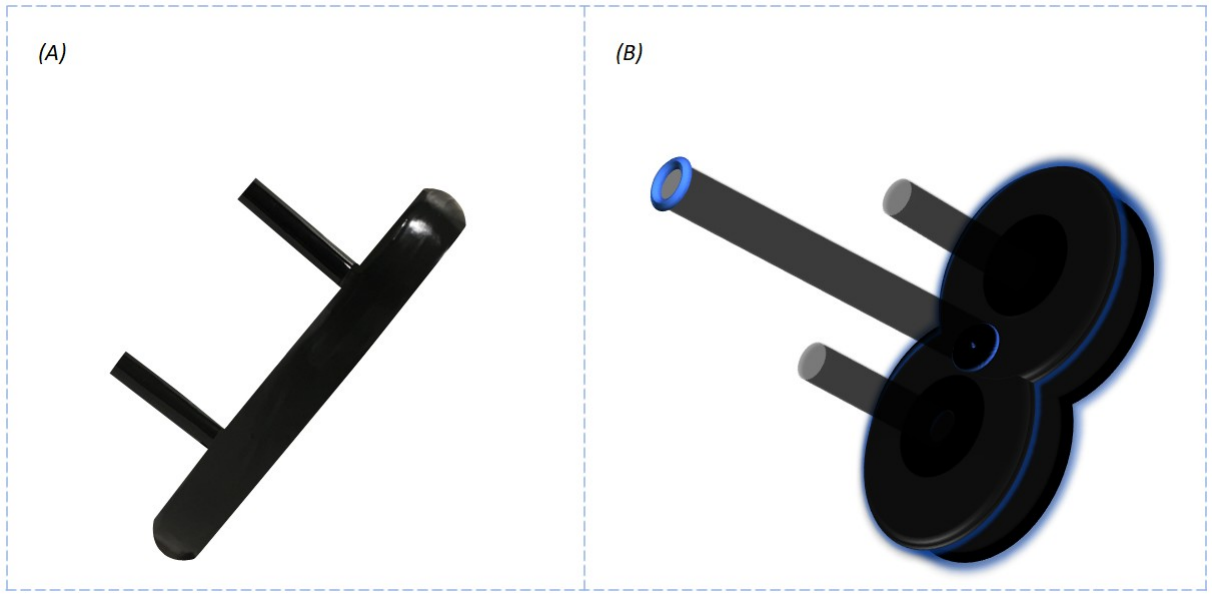
TMS prop. (A) Side view of the plastic prop used to suppress phosphene perception. The props thickness measured 20mm and was wrapped in vinyl tape which was the original encasing of the 40mm figure-of-eight coils. Stemming from the base are two plastic cylinder-shaped bars that were designed to fit tightly within the holes of the coil. (B) Illustration showing the prop mounted onto the coil. The prop was designed to reflect the coils dimensions as seen from this angle.

The other five virtual environments were assigned to the control condition (Fig 4). In order to suppress phosphene perception while replicating other sensory sensations emanating from the 40mm figure-of-eight coils, a plastic prop was designed to replicate the coils physical attributes. This included the coils dimensions and texture which was wrapped in vinyl tape (Fig 5). Therefore, participants could not differentiate between the experimental and control condition through tactile information. Prior to the experiment, all participants went through a screening process to examine the props efficiency. This consisted of evenly randomized trials with and without the prop spanning the experimental design. After ten stimulations, participants were verbally asked to report the sensations they felt, which consisted of the vibrating sensation coming from the coil. If they reported no changes throughout the session, only then were they allowed to continue on with the study. This did not occur on the same day of the actual experiment, but one month prior. Finally, participants were never informed about the control prop during the screening phase and during the day of the experiment. After each condition, the coils were removed and readjusted back onto the head within the same duration to accommodate for the removal of the prop. At a thickness of 20mm, the prop was secured onto the coil and positioned over the predetermined phosphene hotspot [25,30].

## Results and discussion

### Phosphene features

**Fig 6:**
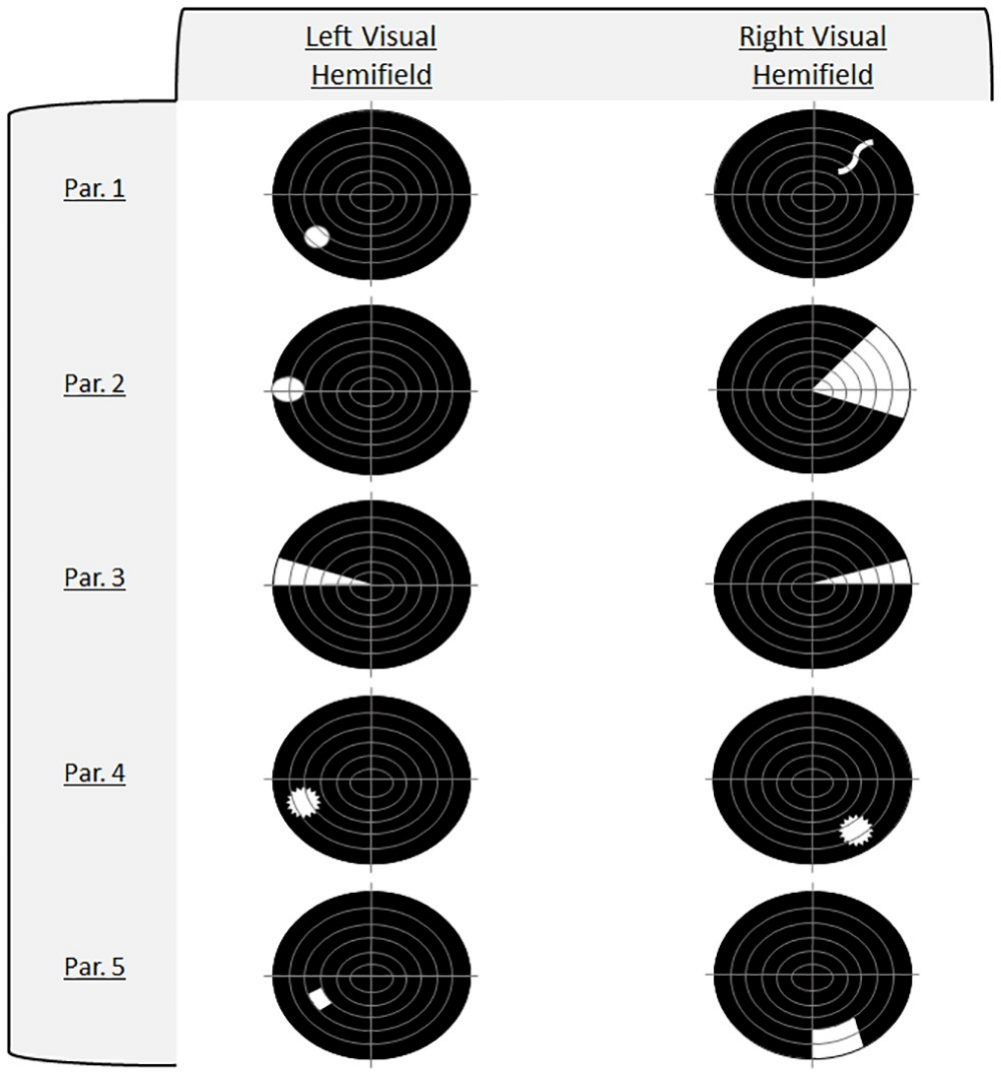
Phosphene features. Visual field plots highlighting phosphene characteristics depicted by each participant.

Participants were required to describe in detail their phosphene characteristics when stimulated over both hemispheres (Fig 6). Phosphene structures included various geometrical shapes and wavy lines. Contralateral stimulation did not result in identical shapes and symmetrical locations, except in participant three, who reported well defined sector-shaped phosphenes in both visual hemifields right above the horizontal meridian. This participant was also the only one to achieve a perfect score. Stimulation sites between both hemispheres occurred 1cm lateral and ranged between 1-4cm dorsal from the inion.

### Navigation precision

Achieving parametric assumptions, a one sample t-test (p < 0.05) was performed to analyze participant’s responses between the control and experimental condition. Cohen’s d was also used to calculate the effect size. In order to determine the reliability of the test given in our experimental design, the Kuder-Richardson Formula 20 (*KR*_20_) was calculated [31]. This formula measures the internal consistency for assessments with dichotomous responses. In this case, assessing participant’s answers in whether or not phosphenes were perceived (yes = 1 and no = 0). This correlation is measured between 0.0 and 1.0, with a value greater than 0.9 indicating a test with high reliability. The test given in our study resulted in a *KR*_20_ value of 0.91.

**Fig 7:**
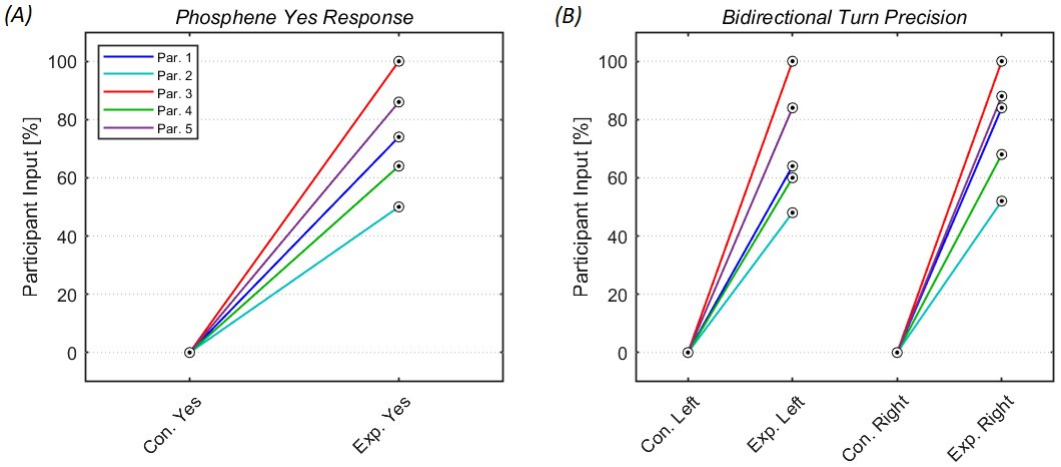
Navigation accuracy. (A) Individual results in response to the first voice-automated question asking participants if they perceived a phosphene. If so, participants will press the right pushbutton indicating “yes.” Phosphenes were only evoked during the experimental condition. (B) Results showing participants ability to accurately turn either left or right based on phosphene awareness.

**Fig 8:**
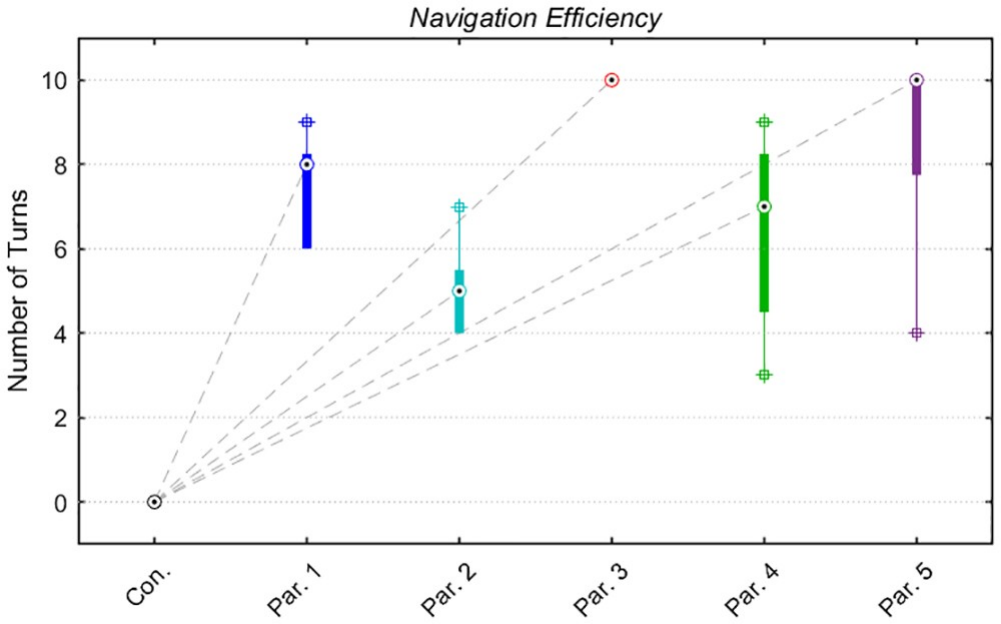
Individual distributions. The boxplot highlights individual distributions needed to advance through all ten turns in the experimental condition. The circles represent the median values for each individual. The bottom edge of the box represents the 25^th^ percentile and the top edge represents the 75^th^ percentile. Lines extending from the box connect to the most extreme values that are not outliers and are represented by crosses. The gray dashed line represents the disparities in median between the control and experimental condition for each participant.

In the control condition, none of the participants answered “yes,” in response to the first voice-automated question. In comparison to the experimental condition, there was a significant difference in participants answering “yes” resulting in an average of 74.8% (SD = 9.66, t(4) = 8.66, p = 0.001, d = 5.46, Fig 7A). Next, the ability to accurately perceive phosphenes in order to turn left or right between the two conditions was calculated (Fig 7B). For both left and right turns in the control condition, the target never advanced past the first turn. Whereas in the experimental condition, the average for initiating left turns was 71.2% (SD = 5.17, t(4) = 7.70, p = 0.0015, d = 4.87) and 78.4% for right turns (SD = 4.67, t(4) = 9.39, p = 0.0007, d = 5.94). The results between experimental right turns and left turns were not significantly different (t(4) = 2.09, p = 0.1045). Overall navigation efficiency varied between participants. The box plot highlights individual distributions to thoroughly exit while advancing successfully past all ten turns (Fig 8).

The last assessment involves mutual information Theory (MI), which measures the dependence of information between two random variables [32]. If two variables (X & Y) are independent, then neither variable contains any information regarding the other. Otherwise, if the variables are equivalent, knowing the entropy of X discloses all information contained in Y. Mutual information was implemented to evaluate the relationship among TMS single pulses and participant’s input based on phosphene awareness facilitating in their ability to advance within the virtual environment. The following equation was utilized to determine the number of bits transferred for each participant:

$$\;I(X;Y)=\sum_{x\in \{r,l,s\}}\sum_{y\in \{r,l\}}P_{\left(X,Y\right)}(x,y)log_{2}(\frac{P_{\left(X,Y\right)}(x,y)}{P_{X}(x)P_{Y}(y)})\;$$

In this case, x is the response made by the participant influencing the direction of the target which can either turn right (r), left (l), or stay (s) at the same location. Variable y represents when a message is being sent, indicating to turn right (r), or left (l). The amount of I is confined within zero and one and expresses the average bit transferred in one turn. Since there are a total of ten turns, multiplying this value by the average gives the sum of bits transferred from the TMS coil to the occipital lobe. Zero bits were transferred in the control condition for all participants since the target remained immobile. The bits transferred in the experimental condition ranged between 5–10, a significant difference between the two conditions (M = 7.55, SD = 1.93, t(4) = 8.72, p = 0.001, d = 5.52, Table 3). Participant z-scores are also shown to highlight individual standard deviations from the average number of total bits transferred in our computer-brain interface (CBI) paradigm (Table 3).

**Table 3:** Bit transfer.

*Participant*	1	2	3	4	5
*Exp*. *Condition*	7.7 (z = 0.08)	5.0 (z = -1.32)	10.0 (z = 1.27)	6.4 (z = -0.59)	8.6 (z = 0.54)

Number of bits calculated for each participant based on the Mutual Information formula in the experimental condition.

In this study, we investigate how computer based virtual environments can be used along with evoking artificial visual inputs in the form of phosphenes to navigate a specified area. Although, our study distinguishes between left and right turns within a computer virtual environment, technological advances in the future could improve phosphene resolution to better project natural conditions. In order to achieve this, probing the neural mechanisms responsible for phosphene perception will become necessary. The effect of TMS over visual areas in order to evoke phosphenes is the result of neuronal excitability [33]. However, the depolarized regions involved in phosphene perception is a highly debated topic. For instance, Salminen-Vaparanta [34] incorporated functional magnetic resonance imaging-guided TMS to understanding the role of visual areas 1 (V1) and 2 (V2). Their results suggest that activation in both areas were capable of producing phosphenes, although stimulation involving V1 generated brighter phosphenes. Kammer [33] also deployed a similar approach and found that the optic radiations situated along the dorsal regions of V1 played a role in evoking phosphenes. The authors also argue that back projecting fibers involving V2 and visual area 3 (V3) regressing towards V1 facilitates in phosphene perception. Finally, Marg and Rudiak [29] concluded that stimulation of subcortical regions were responsible for evoking phosphenes, potentially involving the optic radiation fibers. Romei [35] produced TMS phosphenes while measuring brain activity using electroencephalography (EEG). The researchers showed a relationship between instances of phosphene perception reported by participants with activity measured in the alpha-band (8–14 Hz) within posterior regions. The authors suggest that these changes emulate cortical excitability in visual areas. Another approach to understand the nature of phosphenes requires examining neurochemical interactions. Terhune [36] used magnetic resonance spectroscopy (MRS) in healthy and grapheme-color synesthete participants to investigate the relationship between individual PT values using TMS with glutamate and GABA concentrations in V1. Their results suggest that PT anticipates glutamate levels in V1 and can be attributed to individual distinctions in visual attention. These studies have offered new insights into the neural mechanism facilitating in phosphene perception, however further research is still needed especially when dealing with regions along the visual pathway. Although not reported in our study, TMS over specific visual areas can result in different phosphene colors and even produce moving phosphenes [29]. Understanding the neural basis of these features can be highly beneficial in developing a more sophistical visual prosthesis.

Our results show variations in overall accuracy between participants, while some were able to outperform others. Possible reasons for outcome variability resulting in the turn completion rate of 74.8% (SD = 9.66) could have been due to the absence of implementing a stereotactic guidance system and subtle movements caused by participants. Even the slightest deviation in coil angle or movements of the head has the potential to prevent phosphene perception. Given the binary nature of the questions asked during the experiment, considerations to prevent errors should be evaluated in reference to participants pressing the incorrect button when answering each question. In our experimental design, measures within the computer code were put in place to identify such concerns. However, it is possible that errors within human judgment could impede overall accuracy. Therefore, a visual prosthesis that recognizes such instances will be more persistent at assisting individuals during navigation.

Another important consideration relates to individual changes in PT spanning the experiment. Long term alterations in PT could impinge the feasibility of using this technology as a visual prosthesis. Improving our understanding of the cortical regions responsible for phosphene awareness could greatly increase participant accuracy. A visual prosthesis based on magnetic stimulation would require extreme precision and stability of the coil angle to ensure that the maximum number of bits are transferred. Overcoming these challenges will allow researchers the means to assess the criterion involving PT output based on magnetic stimulation to be considered as a safe and reliable method facilitating in navigation. Finally, current constraints of using a TMS device as a visual prosthesis involves the inability to properly transport it due to its enormous size. Addressing these limitations will be vital if this technology is to become applicable in the future.

One area of research that could solve this problem includes the development of micro-scale magnetic coils, which has been shown to evoke neuronal activity [37–40]. This method also reduces the adverse effects of tissue inflammation characteristic of cortical electrode implants [41]. Such applications would require extensive research in material sciences to position enough magnetic coils over a limited amount of surface area while maintaining enough power to become functional. Although still in its early phases, implementing micro-scale magnetic coils in visual areas to elicit phosphenes might become feasible in the future. Recognizing the side effects of simultaneous activation involving this technology will also need further examination to ensure patient safety. In our study, evoking concurrent phosphenes could have been a viable option to indicate that the target was moving straight. Although, this design was neglected due to safety concerns while using two TMS devices. As a consequence, producing a beep was preferred when the target was advancing to ensure the wellbeing of our participants.

Since visually impaired individuals are the main beneficiaries of this type of research as it relates to navigation, future studies should incorporate this population within the experimental design. The advantage of using virtual environments allows experiments to be conducted in a clinical setting allowing researchers to evaluate and modify any technical flaws within the proposed design. Developing more sophisticated computer simulations to reflect other natural circumstances is a more practical approach at maintaining patient safety while devising effective rehabilitative protocols. Finally, this study also highlights the implications of research surrounding CBI’s. Our CBI paradigm further expands ways to augment sensory information in humans by targeting multiple brain regions, in this case reflecting the topographic organization of the visual system. This method could also be applied to other cortical areas, serving as an alternative solution to potentially counter sensory deficits in certain individuals. On average, 7.55 (SD = 1.93) bits of information was transferred in our CBI setup. As technology improves, these artificial forms of communication could become commonplace leading to a substantial increase in the total bit rate between computers and the human brain.

## Conclusions

The following study investigates the effectiveness of lateralizing phosphenes using TMS over the occipital lobe with the objective of navigating through a virtual environment. The experimental design involved placing two TMS coils over each occipital hemisphere. Instances of phosphene awareness were attributed to the direction where participants were required to turn using two pushbuttons. The objective was to evaluate participants ability to successfully exit each virtual environment relying on lateralized phosphene percepts. Our results show that computer based virtual environments have the potential to assess the effectiveness of a visual prosthesis in facilitating efficient navigation. However, further research is still required to better understand the neural mechanisms involved in phosphene awareness along with the development of new technologies to precisely stimulate visual areas. Both of these considerations will play a substantial role in optimizing overall navigation that can be assessed within computer software’s that are reflective of everyday activities involving blind individuals.

## Supporting information

S1 Table Individual raw data. The following table includes participant responses during the experimental condition. Note that responses for the control condition are not shown since all participants responded “no.” The top section corresponds to the different virtual environments (VE). The number of left (L) and right (R) turns are organized for each participant.
(PDF)

S2 Table Participant information. The table includes those who were able to perceive phosphenes, but fell short of achieving the phosphene threshold required to continue the study.
(PDF)

S3 Table Participant phosphene information. The following tables include the TMS intensities from participants disclosed in S2. The reported TMS intensities reflect the first instance where a phosphene was observed by each participant. Thereafter, TMS intensity output was incrementally increased until reaching the maximum stimulator output. Consequently, since the phosphene threshold criteria was not achieved, these participants were excluded from the study. Finally, intensities for the remaining five participants are not reported since they were not able to perceive any phosphenes.
(PDF)
