# Supplementary material for: Inducing lateralized phosphenes over the occipital lobe using transcranial magnetic stimulation to navigate a virtual environment
Source: PLoS One. 2021 Apr 14;16(4):e0249996. doi: 10.1371/journal.pone.0249996 (PMC8046218; doi:10.1371/journal.pone.0249996)
Supplement: S1 Table — The following table includes participant responses during the experimental condition. Note that responses for the control condition are not shown since all participants responded “no.” The top section corresponds to the different virtual environments (VE). The number of left (L) and right (R) turns are organized for each participant. (PDF) [file pone.0249996.s001.pdf]

Participant	*VE 1*		*VE 2*		*VE 3*		*VE 4*		*VE 5*		*Total*
	L	R	L	R	L	R	L	R	L	R	
1	3	5	5	4	2	6	2	4	4	4	37
2	2	2	2	2	2	3	3	4	3	2	25
3	5	5	5	5	5	5	5	5	5	5	50
4	3	4	3	2	1	2	4	5	4	4	32
5	5	5	5	5	4	5	5	5	2	2	43
