# Supplementary material for: Inducing lateralized phosphenes over the occipital lobe using transcranial magnetic stimulation to navigate a virtual environment
Source: PLoS One. 2021 Apr 14;16(4):e0249996. doi: 10.1371/journal.pone.0249996 (PMC8046218; doi:10.1371/journal.pone.0249996)
Supplement: S2 Table — The table includes those who were able to perceive phosphenes, but fell short of achieving the phosphene threshold required to continue the study. (PDF) [file pone.0249996.s002.pdf]

Participant	*Age*	*Sex*	*Snellen Score*		*Glasses/Onset (Years)*
			Right Eye	*Left Eye*	
6	24	M	20/25	20/25	-
7	22	F	20/50	20/50	+ / 10
8	27	M	20/32	20/32	-
9	26	F	20/100	20/100	+ / 15
10	24	F	20/80	20/63	+ / 14
