# Supplementary material for: Inducing lateralized phosphenes over the occipital lobe using transcranial magnetic stimulation to navigate a virtual environment
Source: PLoS One. 2021 Apr 14;16(4):e0249996. doi: 10.1371/journal.pone.0249996 (PMC8046218; doi:10.1371/journal.pone.0249996)
Supplement: S3 Table — The following tables include the TMS intensities from participants disclosed in S2. The reported TMS intensities reflect the first instance where a phosphene was observed by each participant. Thereafter, TMS intensity output was incrementally increased until reaching the maximum stimulator output. Consequently, since the phosphene threshold criteria was not achieved, these participants were excluded from the study. Finally, intensities for the remaining five participants are not reported since they were not able to perceive any phosphenes. (PDF) [file pone.0249996.s003.pdf]

Left Hemisphere Stimulation:

Participant	*Hotspot Coordinates*	*TMS Intensity*	*Phosphene Shape*	*Phosphene Color*	*Field of Vision*	
					L	*R*
6	F4	54	Circle	White	-	+
7	F5	65	Wavy Line	White	-	+
8	F6	61	Sector	White	-	+
9	F4	58	Circle	White	-	+
10	F5	44	Circle	White	-	+

Right Hemisphere Stimulation:

Participant	*Hotspot Coordinates*	*TMS Intensity*	*Phosphene Shape*	*Phosphene Color*	*Field of Vision*	
					L	*R*
6	H4	59	Sector	White	+	-
7	H6	74	Square	White	+	-
8	H6	68	Arc	White	+	-
9	H4	75	Circle	White	+	-
10	H5	62	Wavy Line	White	+	-
